# Colon perforation by foreign body insertion for sexual gratification: a case report

**DOI:** 10.11604/pamj.2021.40.188.32087

**Published:** 2021-11-29

**Authors:** Alphonce Nsabi Simbila, Ahmed Suphian, Novath Julius Ngowi, Ramadhani Juma Mfinanga, Said Kilindimo, Hendry Robert Sawe

**Affiliations:** 1 Department of Emergency Medicine, Muhimbili National Hospital, Dar es Salaam, Tanzania,; 2 Department of Surgery, Muhimbili University of Health and Allied Sciences, Dar es Salaam, Tanzania

**Keywords:** Rectal foreign body, colon perforation, sexual gratification, auto-eroticism, case report

## Abstract

Occurrence of retained rectal foreign bodies with bowel perforation resulting from auto-eroticism is rare among males in Africa. Embarrassment attached to this condition may delay or derail acquisition of information and management. A 30-year-old male presented with abdominal pain and constipation for 3 days. Abdominal X-rays revealed free air-stripes under both hemidiaphragms and in the peripherals, a 25cm x 5.9cm lucent foreign body on the left side with proximal tapering. There was no evidence of intestinal obstruction. This was consistent with bowel perforation secondary to foreign body introduction. Exploratory laparotomy was performed, a plastic bottle of 250mls was removed from the colon. Transverse repair of a 10cm laceration extending from the rectum to the sigmoid was done and a colostomy placed. A high index of suspicion, a systematic approach and a lower threshold for imaging studies were key to successful management and favorable outcomes of this patient.

## Introduction

Foreign bodies in the rectum can be a result of activities of sexual gratification, psychiatry illness, assault, or iatrogenic introduction during procedure [1]. Given that this condition may result in embarrassment or from psychiatric illness, patients may not be forthcoming and hence health care providers need to have a high index of suspicion during clinical care. Their mere presence in the rectum may not pose any threat or panic to the patient initially, especially when their insertion was for auto-eroticism. Complications such as mucosal lacerations, migration to proximal regions of the intestine, intestinal obstruction, perforations, peritonitis and sepsis can arise from attempts or failure to remove them [2,3]. Majority of these patients first present to the emergency department, so emergency physicians should be systematic in approach and management [4]. Reaching a diagnosis and laying out a management plan can be a challenge for emergency physicians and surgeons. The history given is sometimes vague and inconsistent. A high index of suspicion, privacy, broad-minded history taking, physical examination and choice of investigations are a key to finding out the diagnosis and associated complications early. We present a case of a 30-year-old male with a presumed partial intestinal obstruction due to faecal impaction that turned out to be a rectal foreign body with a colon perforation, requiring surgical intervention.

## Patient and observation

**Patient information:** a 30-year-old male with no significant past medical history presented to the emergency department complaining of abdominal pain and failure to pass stool for 3 days. He had no nausea, vomiting or abdominal distension. He reported passing flatus. Initially, he strongly related his symptoms to inadequate water intake. He later disclosed intentional insertion of a lubricated plastic detergent bottle through the anus for sexual gratification. He endorsed a habit of inserting similar objects through the anus and being able to successfully retrieve them anytime he wished. On this occasion though he lost control of the bottle and it disappeared into the rectum despite his multiple unsuccessful attempts to remove it while at home.

**Clinical findings:** on initial assessment at the emergency department the patient had a blood pressure of 138/86mmHg, heart rate of 89 beats/min, respiratory rate of 19 breaths/min and temperature of 37.5°C. His abdomen was of normal contour, moved with respiration, had a mildly tender left iliac fossa and bowel sounds were normal. There were no features of peritonitis. Digital rectal examination revealed a foreign body 5-6cm distance to the anal verge. The gloved finger was clear.

**Figure 1:**
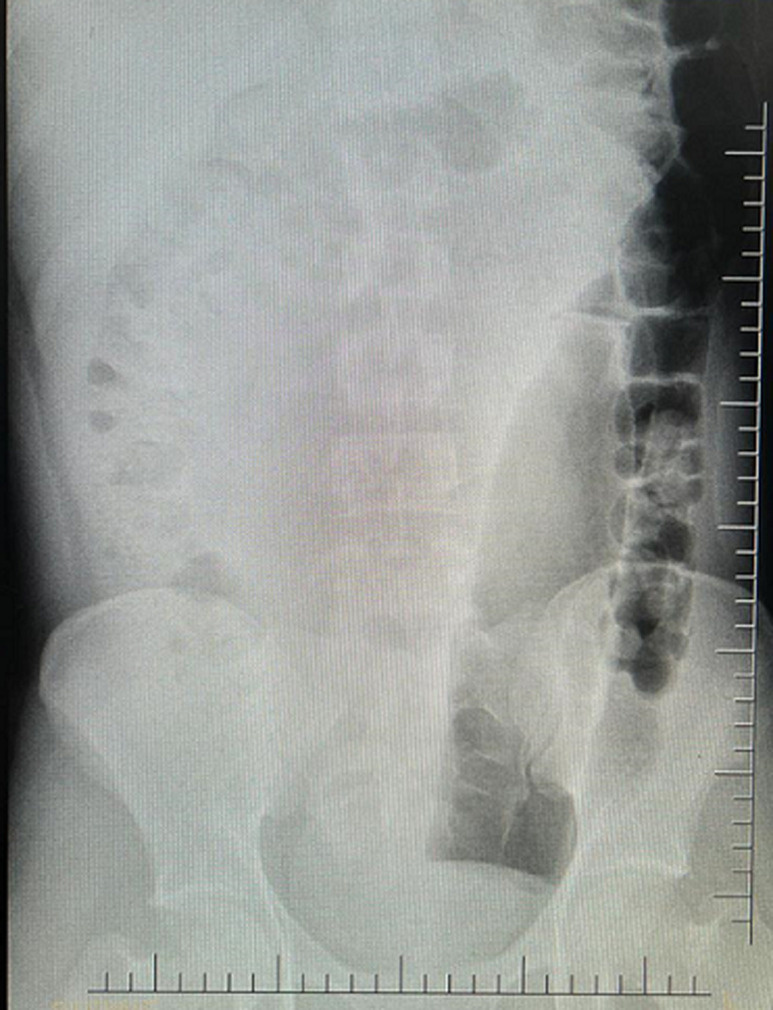
plain abdominal X-ray showing a lucent foreign body on the left side with proximal tapering

**Figure 2:**
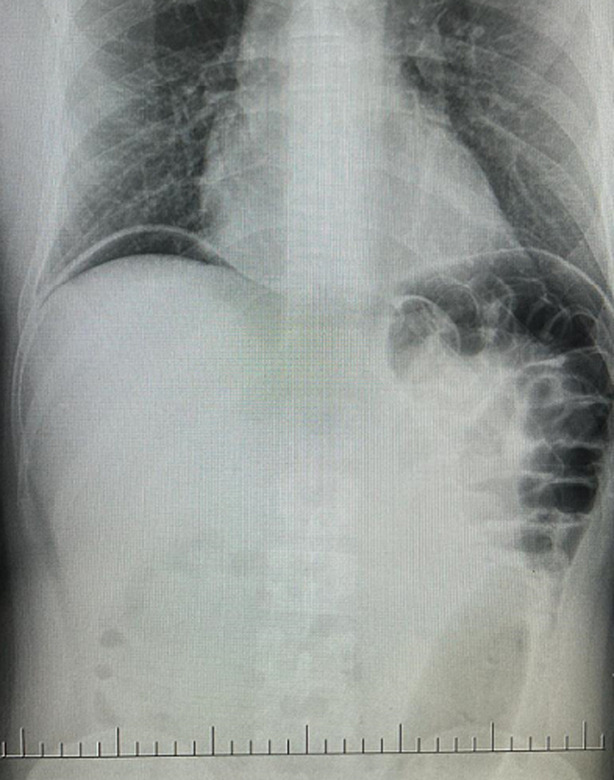
plain abdominal X-rays showing free air-stripes under both hemidiaphragm and in the peripherals

**Diagnostic assessment:** plain abdominal X-rays revealed free air-stripes under both hemidiaphragms and in the peripherals, a lucent foreign body on the left side with proximal tapering measuring 25cm in length and 5.9cm in diameter (Figure 1, Figure 2). There was no evidence of air-fluid levels. Blood samples drawn for point-of-care and other laboratory investigations showed random blood glucose of 6.4mmol/L, potassium of 3.6mmol/L, sodium of 134mmol/L, Chloride of 100mmol/L and lactate of 1.8mmol/L. His full blood count revealed a white cell count of 14,030/µL.

**Diagnosis:** this was consistent with features suggestive of bowel perforation secondary to anal foreign body introduction. Other diagnoses considered were partial intestinal obstruction secondary to faecal impaction, or peritonitis secondary to bowel perforation.

**Figure 3:**
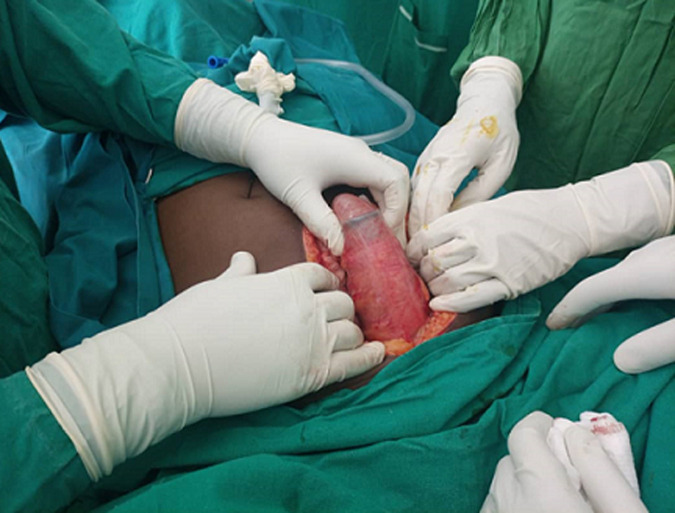
a foreign body (250mls plastic bottle) in the rectum and sigmoid colon

**Figure 4:**
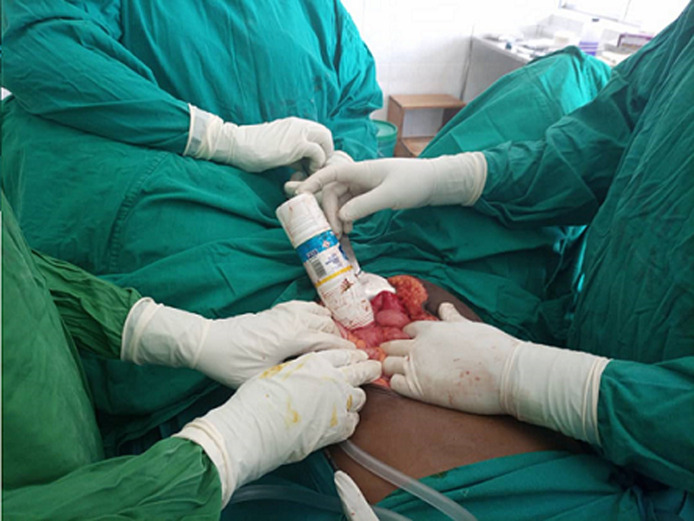
surgical removal of a foreign body (250mls plastic bottle) and a visualized colon laceration

**Therapeutic interventions:** intravenous ceftriaxone (1g stat) and Metronidazole (500mg stat) were initiated. Emergent exploratory laparotomy was performed. A plastic bottle of 250mls was found lying in the rectum and sigmoid colon (Figure 3). There was a 10cm long laceration extending from the rectum to the sigmoid colon. The plastic bottle was removed and transverse repair of the perforation was done (Figure 4). Irrigation of bowels with warm normal saline was done and a proximal colostomy was placed.

**Follow-up and outcome of interventions:** three days of post-operative hospitalization were uneventful. The patient received pain medications, antibiotics, fluids, ulcer prophylaxis and surgical wound care. He was counselled and trained on colostomy use and care. The colostomy was functioning well on the third day and he was discharged from the hospital. At 1 week follow up the patient was doing well with a functioning colostomy. Colostomy reversal was planned after six weeks.

**Patient perspective:** “I didn´t think this illness would be this serious. When I was at home, I tried to fix it myself but realized I wouldn´t succeed. I panicked a lot when the bottle failed to come out and didn´t know what to do until when I was received at the hospital. Thinking about surgery tortured me initially but the doctors explained about the perforation to me. Surgery was the best option for me. I came to terms with it. They even took time to train me on the use and care of the colostomy after surgery. It works fine. It wasn´t easy in the first few days, but I managed, with some adjustments though. I am grateful I landed in safe hands. I resumed my daily routines. I look forward to the colostomy closure. I would like other doctors to learn through my case”.

**Informed consent:** written informed consent was obtained from the patient for publication of this case report, accompanying images and de-identified information.

## Discussion

Abdominal pain is among the commonest presenting complaints at the ED. Some benign presentations of abdominal pain can disguise surgical catastrophes. Physicians are facing an uphill challenge when determining who has or does not have an emergent condition. Foreign bodies placed in the gastrointestinal tract through the mouth are common [5]. However, incidences of foreign bodies inserted through the rectum presenting with intestinal perforation are rare. Majority of cases reported in literature involve middle-aged males with the age ranging between 20 and 40 years [6]. Rectal foreign bodies are an important problem for emergency physicians, whose management options depend on a variety of factors. Physicians working in emergency departments may not think of rectal foreign bodies in patients presenting with abdominal pain in the first place. This is because rectal foreign bodies are either uncommon or under-reported in societies in Africa. This can easily lead to misdiagnosis and management errors. Our patient presented with abdominal pain and failure to pass stool but retained the ability to pass flatus, which initially led the emergency physician to think of partial intestinal obstruction. The type and mode of insertion of rectal foreign bodies differ significantly. Such activities as illegal concealment of illicit drugs by stuffing and assaults have been reported in literature to be among the causes of insertion of rectal foreign bodies [7]. Surrounding cultural norms and practices may make it difficult for male patients who have inserted foreign bodies in the rectum for sexual gratification to be open to health care workers. Literature reports that this practice is common among homosexual men. They usually fabricate stories or attribute their complaint to unnecessary causes. This leads to delayed diagnosis, referral for care and subsequent morbidity and mortality. Our patient gave a history of constipation due to inadequate water intake in an attempt to disguise the aetiology of his condition.

Upon arrival at the emergency department patients are not forthcoming with the possible cause of their complaints. Abdominal pain, anal pain, bleeding from the rectum and failure to pass stool are among the commonest complaints. Among all these presentations our patient had abdominal pain and failure to pass stool only. These findings could easily derail a physician if it weren´t for a high index of suspicion and a broad-minded approach to history taking. Patients present to the emergency department when multiple attempts to remove the foreign bodies fail. These multiple attempts are a potential cause of injuries, migration of the foreign body further up the rectum and perforation of the intestine. Meticulous abdominal examination can reveal signs of peritonitis due to a perforation. A digital rectal examination is helpful in the diagnosis upon palpating a foreign body lodged in the rectum. Our patient attempted to remove the bottle multiple times which could have been the cause of the perforation. He however had no signs of peritonitis upon examination which posed a further challenge to making the right diagnosis [8]. Emergency physicians ought to have a high index of suspicion and instinct. They should maintain a low threshold for obtaining imaging studies once a foreign body is suspected. X-rays of the abdomen and pelvis are of paramount importance in identifying, locating and ruling out intestinal perforation early before manipulation of the foreign body [9]. Our patient had a foreign body in the sigmoid colon with intestinal perforation. These conditions were diagnosed early through a high index of suspicion during history taking and a low threshold to employ imaging studies very early in the management of the patient. Laboratory investigations such as the full blood count play an additional role in circumstances where peritonitis has set in by showing the predominance of white cell counts. Many rectal foreign bodies are manually and successfully removed through the anus after either adequate procedural sedation, local or general anaesthesia depending on the type and circumstances [10]. Laparotomy is the approach of choice in rectal foreign bodies that are impacted or have resulted into a perforation.

## Conclusion

Abdominal pain is a common presentation at acute intake areas. Diagnosing a rectal foreign body resulting from auto-eroticism in males as its aetiology needs a high index of suspicion. A systematic approach ensures early recognition, diagnosis and management plan. Emergency physicians should resist the temptation to manipulate and remove rectal foreign bodies in patients who have had multiple attempts to remove them while at home before ruling out bowel lacerations or perforations. Early use of simple investigations like plain abdominal X-rays are crucial in avoiding pitfalls in the management of rectal foreign bodies and bowel perforations.
